# Structural plasticity of motor cortices assessed by voxel-based morphometry and immunohistochemical analysis following internal capsular infarcts in macaque monkeys

**DOI:** 10.1093/texcom/tgac046

**Published:** 2022-11-08

**Authors:** Kohei Matsuda, Kazuaki Nagasaka, Junpei Kato, Ichiro Takashima, Noriyuki Higo

**Affiliations:** Human Informatics and Interaction Research Institute, National Institute of Advanced Industrial Science and Technology (AIST), 1-1-1 Umezono, Tsukuba, Ibaraki 3058568, Japan; Graduate School of Comprehensive Human Sciences, University of Tsukuba, Ibaraki 3058577, Japan; Human Informatics and Interaction Research Institute, National Institute of Advanced Industrial Science and Technology (AIST), 1-1-1 Umezono, Tsukuba, Ibaraki 3058568, Japan; Institute for Human Movement and Medical Sciences, Niigata University of Health and Welfare, Niigata 9503198, Japan; Department of Physical Therapy, Faculty of Rehabilitation, Niigata University of Health and Welfare, Niigata 9503198, Japan; Human Informatics and Interaction Research Institute, National Institute of Advanced Industrial Science and Technology (AIST), 1-1-1 Umezono, Tsukuba, Ibaraki 3058568, Japan; Faculty of Medicine, University of Tsukuba, Ibaraki 3058577, Japan; Human Informatics and Interaction Research Institute, National Institute of Advanced Industrial Science and Technology (AIST), 1-1-1 Umezono, Tsukuba, Ibaraki 3058568, Japan; Graduate School of Comprehensive Human Sciences, University of Tsukuba, Ibaraki 3058577, Japan; Human Informatics and Interaction Research Institute, National Institute of Advanced Industrial Science and Technology (AIST), 1-1-1 Umezono, Tsukuba, Ibaraki 3058568, Japan

**Keywords:** diaschisis, gray matter volume, macaque model, structural compensation, ventral premotor cortex

## Abstract

Compensatory plastic changes in the remaining intact brain regions are supposedly involved in functional recovery following stroke. Previously, a compensatory increase in cortical activation occurred in the ventral premotor cortex (PMv), which contributed to the recovery of dexterous hand movement in a macaque model of unilateral internal capsular infarcts. Herein, we investigated the structural plastic changes underlying functional changes together with voxel-based morphometry (VBM) analysis of magnetic resonance imaging data and immunohistochemical analysis using SMI-32 antibody in a macaque model. Unilateral internal capsular infarcts were pharmacologically induced in 5 macaques, and another 5 macaques were used as intact controls for immunohistochemical analysis. Three months post infarcts, we observed significant increases in the gray matter volume (GMV) and the dendritic arborization of layer V pyramidal neurons in the contralesional rostral PMv (F5) as well as the primary motor cortex (M1). The histological analysis revealed shrinkage of neuronal soma and dendrites in the ipsilesional M1 and several premotor cortices, despite not always detecting GMV reduction by VBM analysis. In conclusion, compensatory structural changes occur in the contralesional F5 and M1 during motor recovery following internal capsular infarcts, and the dendritic growth of pyramidal neurons is partially correlated with GMV increase.

## Introduction

There are reports on compensatory plastic changes in the remaining intact brain regions during motor function recovery following stroke, which typically occurs several months after onset (Nakayama et al. 1994; Smith et al. 2017). Functional brain imaging has revealed compensatory activation during movements following recovery (Johansen-Berg et al. 2002; Schaechter and Perdue 2008; Bestmann et al. 2010; Rehme et al. 2011; Bradnam et al. 2012; Kantak et al. 2012; Bajaj et al. 2016; Touvykine et al. 2016; Dodd et al. 2017); however, the mechanism by which structural plastic changes underlie functional reorganization is unclear. Several studies have assessed changes of the cerebral gray matter volume (GMV) by a voxel-based morphometry (VBM) analysis of magnetic resonance imaging (MRI) data following stroke (Dang et al. 2013; Fan et al. 2013; Abela et al. 2015; Cai et al. 2016; Jiang et al. 2017; Wilkins et al. 2017; Yu et al. 2017; Miao et al. 2018; Wang et al. 2018; Chen et al. 2021), for review see (Cortese et al. 2021). They reported a significant GMV increase in widespread areas involved in motor, cognitive, emotional, or somatosensory functions such as the primary motor cortex, supplementary motor cortex, caudate nucleus, cerebellum, frontal cortex, hippocampus, precuneus, orbitofrontal cortex, cingulate cortex, and thalamus in the hemispheres both ipsilateral and contralateral to the stroke lesion; nonetheless, there is no consensus on increased GMV responsible for motor function recovery. This may be attributed to the variance in patient characteristics, such as periods from stroke onset and the size and location of the stroke lesions.

Thus, we performed a VBM analysis in a macaque model of unilateral internal capsular infarcts (Murata and Higo 2016). Brain imaging with functional near-infrared spectroscopy revealed that increased activation in the ventral premotor cortex is involved in the recovery of dexterous hand movement post infarction (Kato et al. 2020). VBM analysis in this model will elucidate GMV changes associated with motor recovery following stroke to the internal capsule (IC), a brain region specifically dedicated to motor function.

In addition, the cellular basis of GMV changes is unclear. Despite the correlation between GMV changes and changes in cell density (Suzuki et al. 2013) and dendritic morphology (Kassem et al. 2013) in rodents, it is essential to apply the knowledge from studies in nonhuman primates to human patients. This is because rodents are different from primates in terms of their brain architecture and responses to brain damage. For instance, the motor pathways of some primate species, including humans and macaques, but not rodents, are directly connected to spinal motor neurons (Lemon and Griffiths 2005; Courtine et al. 2007) and hierarchically organized motor cortical areas (Rizzolatti and Luppino 2001; Tanji 2001; Dum and Strick 2002) to finely control their digits and limbs. Moreover, the time course of the proliferation of microglia and their function following brain damage differ between primates and rodents (Thiel et al. 2010; Wang et al. 2013; Nagasaka et al. 2017; Yeo et al. 2019; Yew et al. 2019; Kato et al. 2021). Therefore, histological analyses combined with VBM analysis in the macaque model of IC infarcts will provide useful information on structural plastic changes that occur during functional recovery following stroke in primate brains. To address the cellular basis underlying the GMV changes, we intended to perform immunohistochemistry (IHC) in cortical sections obtained from macaques used for VBM and to investigate changes in cell density and dendritic morphology using an SMI-32 antibody (neurofilament antibody), which specifically stains pyramidal neurons.

## Materials and methods

### Study animals

**Fig. 1:**
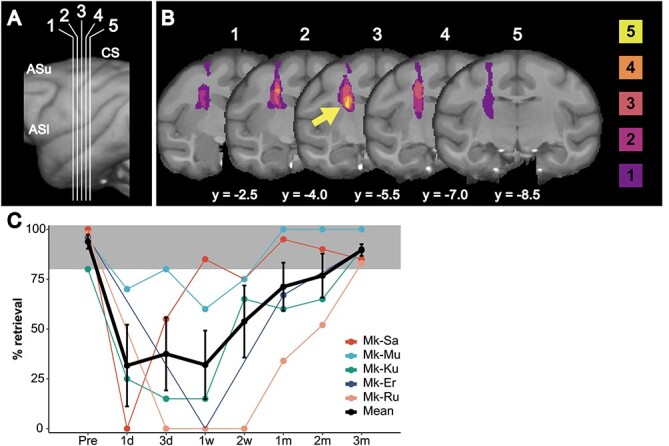
Lesion distribution (A, B) and behavioral change (C). A) A macaque brain template image in which vertical white lines indicate the location of the coronal slices displayed in B. B) Lesion mapping on the T2-weighted image normalized to the macaque brain template. The arrow indicates the posterior IC where the lesion was found in all of the macaques examined. The colors of each cluster indicate the number of macaques with lesions in the indicated voxels. C) The success rate of the vertical slit task increased at 2 weeks following infarcts and then reached over 80% (the filled area) at 3 months after onset. The black line represents the mean ± SEM. CS, central sulcus; ASu, upper limb of the arcuate sulcus; Asl, lower limb of the arcuate sulcus.

We used 10 macaques (*Macaca fuscata*, 4 males, 4.2–8.8 kg, aged >4 years) in this study (Table 1). Five macaques (2 males, 4.5–8.8 kg) underwent VBM analysis, where unilateral IC infarction was induced by injecting endothelin-1 into the posterior IC, a peptide that causes vasoconstriction (Yanagisawa et al. 1988). The lesioned macaques were the same as those used in our previous studies (Murata and Higo 2016; Kato et al. 2020). The volume and temporal profile of the lesion, as well as behavioral recovery, have already been reported in previous studies. Using the data obtained in these studies, the present study also showed the lesion extent, which is overlaid on a template image (Fig. 1B). Brain sections were obtained from the lesioned macaques and 5 intact macaques (2 males, 4.2–8.2 kg), 2 of which received endothelin-1 injection and were immediately perfused for immunohistochemical analysis. Mann–Whitney test did not reveal a statistically significant difference in the body weight (*P* = 0.84) between the lesioned and intact macaques. The macaques were either purchased from a local provider (Kawahara Bird-Animal Trading Go Ltd, Tokyo, Japan) or bred at the Primate Research Institute of the Kyoto University. The current study was approved by the Animal Research Committee of the National Institute of Advanced Industrial Science and Technology and conformed to the NIH Guidelines for the Care and Use of Laboratory Animals. To minimize daily stress on macaques, we controlled the humidity, temperature, and light in the room, besides providing amenities, such as toys. Fresh fruits and vegetables were provided daily, and feeding was tailored to their individual preferences and body conditions. To ensure health management, changes in physical condition were monitored daily by the researchers and laboratory staff.

**Table 1:** Macaques used in the study.

	Sex	Weight (kg)	Survival period after injection	Infarct volume at 1 month following injection (mm^3^) (within the IC)
Mk-Sa	Male	5.2	3 months	163.5 (102.2)
Mk-Mu	Female	4.5	3 months	175.5 (139.5)
Mk-Ku	Male	8.8	3 months	157.5 (83.8)
Mk-Er	Female	5.0	6 months	91.2 (38.4)
Mk-Ru	Female	6.8	6 months	117.6 (117.6)
Mk-BM	Female	5.3	Intact (no injection)	
Mk-Ok	Female	8.0	Intact (no injection)	
Mk-Ak	Female	8.2	Intact (no injection)	
Mk-As	Male	6.3	Intact (0 day)	
Mk-Bl	Male	4.2	Intact (0 day)	

### Induction of IC infarction

Unilateral IC infarction was induced using a previously described procedure (Murata and Higo 2016). Briefly, we determined the preferred hand of the macaques by identifying the hand used to reach and grasp the target object (Murata et al. 2008; Murata et al. 2015). The location of the posterior IC was identified using 3.0 Tesla MRI systems (3T Signa LX, General Electric Medical Systems, Milwaukee, WI, or Philips Ingenia 3.0T, Philips Healthcare, Best, the Netherlands). Before the scan, the macaques were anesthetized with medetomidine (0.05 mg/kg), midazolam (0.3 mg/kg), and ketamine (4 mg/kg). Specifically, MR images were taken from animals that were fixed to an MRI-compatible stereotaxic frame. Thereafter, stereotaxic coordinates of the posterior IC, where the neuronal pathways originating from the hand area of the primary motor cortex (M1) passed through the hemisphere contralateral to the preferred hand, were determined on the basis of data from anatomical tracer studies (Morecraft et al. 2002; Schmahmann and Pandya 2009). The injections of endothelin-1 (1.5 μg/μL, 4198-v, Peptide Institute, Inc., Osaka, Japan) were also performed using a stereotaxic frame, in which the microsyringe was directed vertically downward toward the stereotaxic coordinates after craniotomy over the IC under pentobarbital anesthesia (25 mg/kg). A total of 15 injections were administered; 3 depths at 1.5 mm intervals for each of the 5 injections in a rostrocaudal direction, centered at the identified stereotaxic coordinates of the posterior IC. At each site, 8 μL of endothelin-1 was injected at a rate of 2 μL/min. We calculated spatiotemporal changes in the infarct volume on T2-weighted MRI using Stereo Investigator imaging software (MBF Bioscience, Williston, VT, USA). For lesion mapping on MRI, individual T2-weighted images were normalized and overlaid to a macaque template (Rohlfing et al. 2012) using SPM12 (https://www.fil.ion.ucl.ac.uk/spm/software/spm12/) run in MATLAB (MathWorks Inv., Sherborn, MA). Subsequently, tissues other than the brain were stripped using FSL (https://fsl.fmrib.ox.ac.uk/fsl/fslwiki/FSL). MRIcron (https://www.nitrc.org/projects/mricron/) and MRIcroGL (https://www.nitrc.org/projects/mricrogl/) were used for delineating the lesion extension and for displaying lesion mapping on the template, respectively.

### Behavioral assessment

As performed in our previous study (Murata et al. 2008), vertical slit task was used for evaluating the performance of hand movement before and at the following time points after infarction: 1 day, 3 days, 1 week, 2 weeks, 1 month, 2 months, and 3 months. In this task, macaques were instructed to retrieve a small food pellet (7 × 7 × 7 mm in size) inserted into the vertical slit (10 mm in width); 20 trials in a day at 3 times within a week. Failure of the task was defined as follows: food pellet slipping off their hand, use of non-affected hand (non-preferred hand), and no grasping over 20 s since food pellet was provided.

### Voxel-based morphometry

**Fig. 2:**
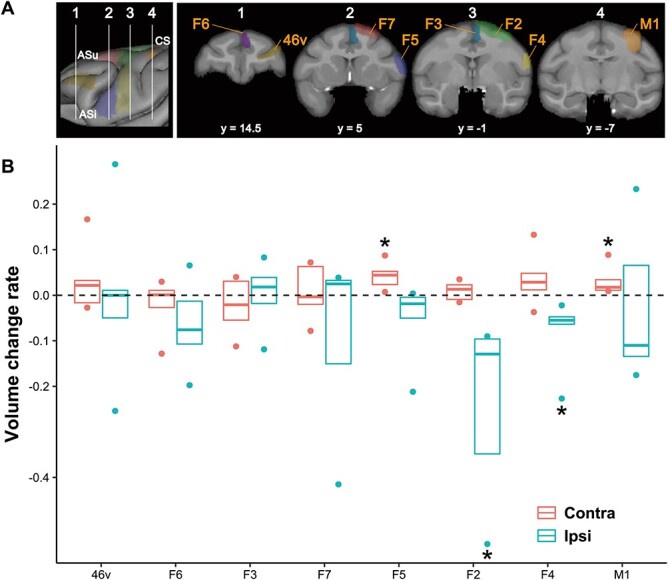
GMV change rate 3 months after infarcts in each ROI for 8 cortical areas. A) Vertical white lines in the left panel indicate the location of the coronal slices displayed in the right panel, comprising the ROIs in the 46v (mustard yellow), F6 (purple), F3 (blue-green), F7 (red), F5 (blue), F2 (green), F4 (yellow), and M1 (orange). B) GMV change rate 3 months after stroke normalized by GMV 1 week after infarcts. The one-sample Wilcoxon signed-rank test (one-sided) showed a significant GMV increase in the contra-F5 and contra-M1, but decrease in the ipsi-F2 and ipsi-F4 relative to the baseline (GMV 1 week after infarcts) (*P < 0.05). The horizontal line in each boxplot represents the median value, and the top and bottom of the box correspond to the 75th and 25th percentile values, respectively. The dots represent the maximum and minimum values in each area. 46v, ventral prefrontal cortex; F6, pre-supplementary motor area; F3, supplementary motor area; F7, dorsal–rostral part of the premotor cortex; F5, ventral–rostral part of the premotor cortex; F2, dorsal–caudal part of the premotor cortex; F4, ventral–caudal part of the premotor cortex; M1, hand area of primary motor cortex; CS, central sulcus; ASu, upper limb of the arcuate sulcus; Asl, lower limb of the arcuate sulcus.

The T1-weighted images of each lesioned macaque at prelesion, 1 week, 2 weeks, 1 month, 2 months, and 3 months following infarction were used for VBM analysis with SPM12. First, we removed the tissues other than the brain from T1-weighted images using the brain extraction tool in Mango (http://ric.uthscsa.edu/mango/plugin_jbet.html) and adjusted the lesioned side to the left. All images were manually reoriented. Subsequently, we separated the macaque brain tissue into gray matter (GM), white matter, and cerebrospinal fluid based on the probability maps provided by Rohlfing et al. (2012). A customized GM template was constructed from the pre-lesion scan based on Diffeomorphic Anatomical Registration using Exponentiated Lie Algebra. After spatially normalizing individual GM images to the customized template, the images were registered to an INIA19 macaque template with 0.5 × 0.5 × 0.5 mm in voxel size. We applied Jacobian determinants to the voxel value modulation to compare the actual GM volume corrected by the total volume for each individual. Subsequently, we performed smoothing using a Gaussian filter with a 2 mm full-width at half-maximum. To exclude the white matter and cerebrospinal fluid from the statistical analysis, automatic mask creation was conducted using the SPM masking toolbox (http://www0.cs.ucl.ac.uk/staff/g.ridgway/masking/). For the statistical analysis, we first performed a ROI-based analysis to investigate longitudinal GMV changes in the motor-related cortices expected to be susceptible to the infarction as follows: supplementary motor area (F3), dorsal–rostral (F7), dorsal–caudal (F2), ventral–rostral (F5), ventral–caudal (F4) premotor cortices, and M1. Additionally, as control regions, we also set region of interest (ROI) in the ventral prefrontal cortex (46v) and pre-supplementary motor area (F6), which are mainly involved in cognitive function (Fig. 2A) (Nelson and Guyer 2011; Ruan et al. 2018). Each anatomical position of the ROI was identified based on the 3-D atlas of the macaque brain (Reveley et al. 2017). For M1, the ROI was positioned in the hand area, defined as the area located between the caudal end of the precentral dimple and the central sulcus of coronal images through the superior genu of the central sulcus (Murata et al. 2015; Higo et al. 2016). In the ROI-based analysis, GMV 3 months after infarcts was normalized by the GMV 1 week after infarcts when primary behavioral recovery occurred, and then compared to the baseline statistically. We next performed the whole-brain analysis, in which the nonparametric *t*-test was conducted to compare the GMV changes in each voxel of the whole brain between 1 week and 3 months post infarcts using the statistical nonparametric mapping toolbox (https://warwick.ac.uk/fac/sci/statistics/staff/academic-research/nichols/software/snpm/). The cluster-defining threshold was set to *P*_uncorrected_ < 0.001 (*T* = 7.1723), and family-wise error (FWE) correction was applied to cluster-wise threshold at *P*_corrected_ < 0.05 (*k* = 111) (Quallo et al. 2009; Cooper et al. 2021).

### Brain tissue preparation

Brain tissues were obtained from 5 lesioned macaques at 3 or 6 months following infarction and 5 intact macaques (Table 1) (Higo et al. 1998, 2002a, 2002b). The macaques were anesthetized with pentobarbital sodium (35–50 mg/kg) and perfused through the ascending aorta with ice-cold saline (0.5 L) containing sodium heparin (1000 units/mL), followed by an ice-cold fixative consisting of 4% paraformaldehyde and 0.1% glutaraldehyde in phosphate buffer (pH 7.4). Following perfusion, the brain was immediately removed and blocked in the coronal plane (5 mm thick). Subsequently, the blocks were immersed in a post-fixative solution containing 2% paraformaldehyde/5% sucrose in phosphate buffer, followed by successive immersions in phosphate buffer containing 10%, 20%, and 30% sucrose. Thereafter, the brain blocks were mounted in an optimal cutting temperature compound (Miles, Inc., Elkhart, IN, USA), rapidly frozen in a dry ice–acetone bath, and stored at −80 °C until dissection. We prepared 16-μm-thick coronal sections from the motor cortices using a cryostat (NX70, Thermo Fisher Scientific, MA), and the sliced sections were attached to a glass slide (Matsunami Glass Inc., Japan). Each motor cortical area was identified by sulcal landmarks and cytoarchitectures, which were visualized by Nissl staining of adjacent sections, with reference to neuroanatomical studies (Matelli et al. 1985; Saleem and Logothetis 2006).

### IHC and quantification

We performed IHC using the monoclonal mouse SMI-32 antibody (1:1000, BioLegend, San Diego, CA, USA, Cat# 801701, RRID: AB_2564642) (Yamamoto et al. 2013) to visualize dendrites in the motor cortices where significant or large GMV changes were indicated in the region of interest (ROI)-based analysis, i.e. F7, F2, F5, F4, and M1. The immune complex was visualized by an avidin–biotin–peroxidase method using Vectastain Elite ABC kits (Vector Laboratories, Inc., Burlingame, CA, USA). For IHC, we analyzed 2 SMI-32-labeled sections that were taken from ROIs in the 5 motor cortices, as shown in Fig. 2A, per hemisphere per individual. Subsequently, we quantified the density and the dendritic arborization of SMI-32-positive pyramidal neurons in layer V, which was determined using adjacent Nissl-stained sections in reference to a cytoarchitectonic atlas (Saleem and Logothetis 2006). For the cell density analysis, images were captured using a microscope (BX63, Olympus, Tokyo, Japan) equipped with a 3-charge-coupled device color video camera (DV-47d, MBF Bioscience). SMI-32-positive neurons were counted in the 200-μm-thick ROI, which is required for covering the majority of neurons in the layer in the macaque cortex (Hutsler et al. 2005), in layer V within a 3-mm column vertical to the cortical surface in each motor cortex using Neurolucida 9 software (MBF Bioscience). We calculated the cell density (cells/mm^2^) by dividing the number of SMI-32-positive neurons by the area of the ROI (mm^2^). For dendritic arborization analysis, 6 pyramidal neurons with clearly visible dendrites were randomly selected per section to cover the ROI on the section from medial to lateral; those with overlapping cell bodies or segmented dendrites were excluded. Subsequently, we performed Sholl analysis as described in a previous study (Contestabile et al. 2018). In this analysis, a series of concentric circles were centered on the cell body at 10-μm intervals using Neurolucida software, and the number of intersections between the dendritic branches and circles was counted. Moreover, we calculated the total number of intersections per neuron.

### Statistical analysis

We used R version 4.1.2, namely R studio version 1.4.1717 (https://www.r-project.org/) for the statistical analysis, in which parametric and nonparametric tests were applied to the present data with sample sizes of over and less than 30, respectively, as a sample size of 30 is considered to be a border for the central limit theorem (Kwak and Kim 2017). To determine the *P*-values, we performed paired or nonpaired *t*-tests corrected by the Bonferroni method for comparing the total number of dendritic intersections among the paired group (ipsilesional [ipsi]/contralesional [contra]) and nonpaired group (intact/ipsi and intact/contra), respectively, in which intact indicates the data obtained from both the left and right hemispheres of the intact macaques per region, using the pairwise_t_test() function from the rstatix package. In addition, we applied the 2-way factorial analysis of variance (ANOVA) with multiple pairwise comparisons corrected by the Bonferroni method of the estimated mean to the comparison of the number of dendritic intersections, depending on the distance from the soma (10–250 μm) between each hemisphere, intact/ipsi and intact/contra, in the lesioned macaques as well as the merged data for individuals per region using the emmeans_test() function from the rstatix package. Paired *t*-test was conducted for the analysis of dendritic arborization depending on the distance from the soma between hemispheres per intact macaque using the pairwise_t_test() function from the rstatix package. To determine significant differences in the cell density, Wilcoxon signed rank test or Wilcoxon rank sum test corrected by the Bonferroni method was performed for the paired group (ipsi/contra) and nonpaired group (intact/ipsi and intact/contra) using the pairwise_wilcox_test() function from the rstatix package. To identify significant differences in longitudinal GMV changes in each ROI between 3 months and 1 week after infarcts, we conducted the one-sample Wilcoxon signed-rank test (one-sided) using the wilcox_test() function from the rstatix package. We performed a regression analysis with Pearson correlation coefficients to elucidate the relationship between lesion volume within the IC and both GMV change rate and behavioral performance and between GMV and both the total number of dendritic intersections and cell density using the geom_smooth() and stat_cor() function in the ggplot2 and ggpubr package, respectively. The GMV change rate was computed using the following formula [(3m–1w)/((3m + 1w) × 0.5)] where 3m and 1w indicated GMV at 3 months and 1 week following infarcts, respectively (Hopkins et al. 2017). To calculate the effect size, we determined the Cohen’s *d* (*d*) in cohens_d() function and *r* in wilcox_effsize() function for parametric/nonparametric multiple comparisons, respectively.

## Results

### GMV changes

On the day following the infarcts in the IC, macaques displayed upper-limb paralysis contralateral to the lesioned hemisphere, which gradually recovered from 1 week to 3 months (Fig. 1C), as previously reported (Murata and Higo 2016; Kato et al. 2020). The cerebral edema expanded outside the posterior IC 3 days post infarction when the volume peaked, whereas the lesion was highly localized within the posterior IC from 2 weeks onwards (Fig. 1A and B), as described in a previous report (Murata and Higo 2016). In this study, we further identified lesion overlay and investigated structural cerebral changes and the cellular basis underlying the changes during recovery periods (from 1 week after infarcts) together with VBM analysis and IHC with SMI-32 antibody.

**Fig. 3:**
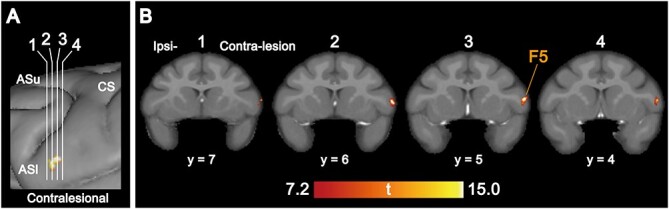
Brain regions displaying a significant GMV increase post infarction. A) Vertical white lines indicate the location of the coronal slices displayed in B. B) Significant GMV increase is observed in the contra-F5 as one consecutive cluster with a maximum at the third slice (*x*, contra-ipsilateral = 24.0, *y*, rostrocaudal = 5.0, and *z*, dorsoventral = 4.0). The cluster-defining threshold is set at *P*_uncorrected_ < 0.001, with an extent threshold P_corrected_ < 0.05 (FWE correction). The scale bar indicates the *T* score. F5, ventral–rostral part of the premotor cortex; CS, central sulcus; ASu, upper limb of the arcuate sulcus; ASl, lower limb of the arcuate sulcus.

Representative lesion locations at 1 month after infarcts are shown in Fig. 1A and B, in which the arrow indicates the lesion overlay with the maximum number of animals. ROI-based analysis between 1 week and 3 months after infarction showed a significant GMV increase in both the contra-F5 (*P* = 0.0312) and contra-M1 (*P* = 0.0312), and a significant decrease in both the ipsi-F2 (*P* = 0.0312) and ipsi-F4 (*P* = 0.0312) (Fig. 2B). Whole-brain analysis revealed a single cluster located in the F5 of the contra-hemisphere (*k* = 150), with increased GMV at 3 months than that of 1 week post infarcts (cluster-level *P*-value at P_corrected_ = 0.0313); a maximum difference at the following coordinates (*x* = 24.0, *y* = 5.0, and *z* = 4.0, Fig. 3) was observed. No regions displayed significant GMV decreases in the whole-brain analysis.

**Fig. 4:**
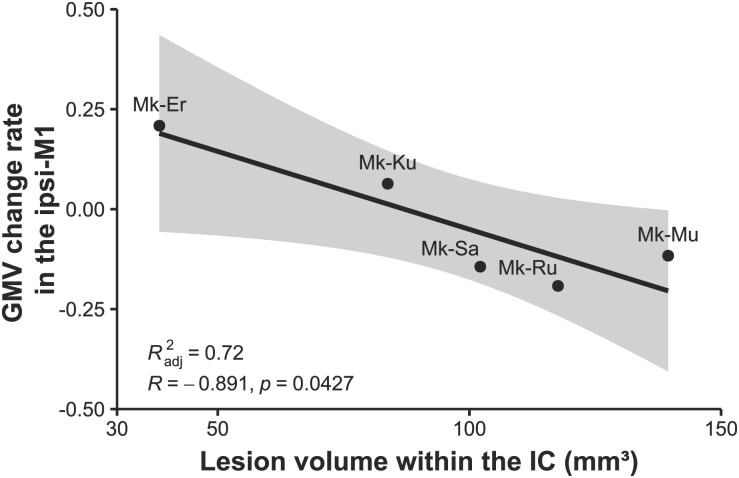
Correlation between the lesion volume within the IC and GMV changes in the ipsi-M1. The lesion volume within the IC 1 month post infarcts is negatively correlated with GMV changes in the ipsi-M1. For statistical analysis, the regression analysis with Pearson correlation coefficients has been performed. Shaded area indicates 95% confidence interval. M1, hand area of primary motor cortex.

We expected a decrease in GMV in the hand area of the ipsi-M1 because retrograde atrophy or degeneration presumably occurs in the motor projection neurons that send projections to the IC; nonetheless, no significant changes were detected in the ipsi-M1. However, a correlation analysis between the IC lesion volume and GMV changes in the ipsi-M1 indicated a significant negative correlation (*R*^2^ = 0.72, *R* = −0.891, and *P* = 0.0427, Fig. 4), suggesting that retrograde atrophy or degeneration depended on the size of the lesion in the IC. The retrograde atrophy or degeneration induced by the IC lesion in the ipsi-M1 is thought to negatively affect motor performance; the lesion volume in the IC had a negative correlation trend with the success rate of precision grip using the tips of the thumb and first finger, although not significant (*R*^2^ = 0.50, *R* = −0.792, and *P* = 0.11, data not shown).

### Histological changes

**Fig. 5:**
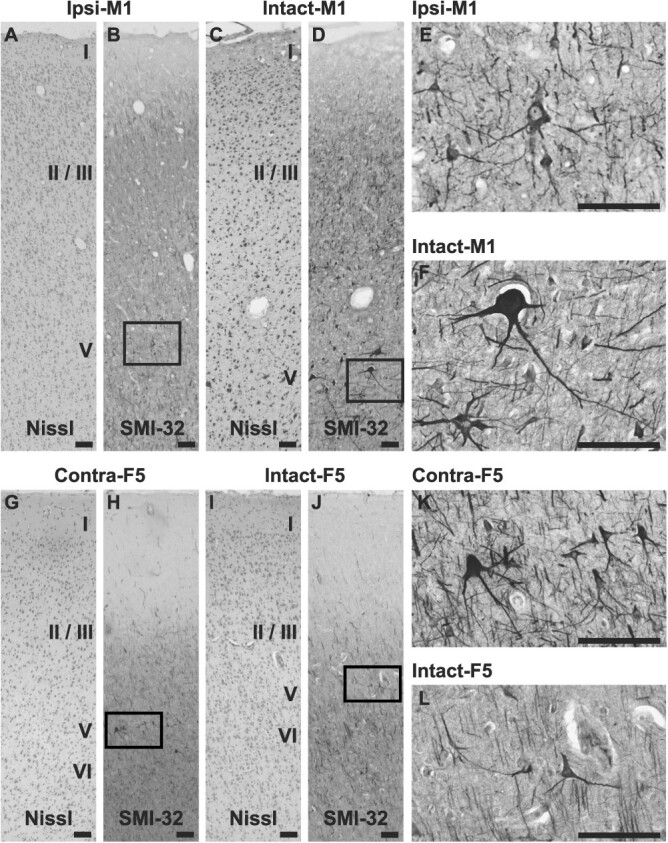
IHC using SMI-32 antibody in M1 (A–F) and F5 (G–L). A, C, G, and I) Nissl-stained sections adjacent to the SMI-32 sections displayed in B, D, H, and J. E, F, K, and L) High magnification images of the pyramidal neurons in the layer V within the squares in B, D, H, and J, respectively. SMI-32-positive pyramidal neurons with smaller cell body are frequently observed in the layer V in the hand area of ipsi-M1, relative to that in the M1 of intact macaques. Contrarily, the neurons with extensive arborization are more frequently observed in the contra-F5 than in the intact F5. M1, primary motor cortex; F5, ventral–rostral part of the premotor cortex. Scale bars: 100 μm.

To clarify cellular basis of underlying GMV changes, we conducted immunohistochemical analysis in the brain regions including the F7, F2, F5, F4, and M1, where significant or large GMV changes were indicated in ROI-based analysis. Although in the macaques used in the histological analysis, the time since lesion varied as shown in Table 1, no correlation was found between time after lesion and the number of dendritic intersections in any region examined (ipsi-F7, *P* = 0.15; ipsi-F2, *P* = 0.45; ipsi-F5, *P* = 0.18; ipsi-F4, *P* = 0.96; ipsi-M1, *P* = 0.43; contra-F7, *P* = 0.32; contra-F2, *P* = 0.70; contra-F5, *P* = 0.22; contra-F4, *P* = 0.80; contra-M1, *P* = 0.073), suggesting that histological changes were completed by approximately 3 months after infarcts. The histological analysis revealed smaller neuronal cell bodies of SMI-32-positive pyramidal neurons in the layer V in the hand area of the ipsi-M1, relative to those in the M1 area of intact macaques (Fig. 5A–F). In contrast, SMI-32-positive neurons with extensive arborization were more frequently observed in the contra-F5 than that in the intact F5 (Fig. 5G–L).

**Fig. 6:**
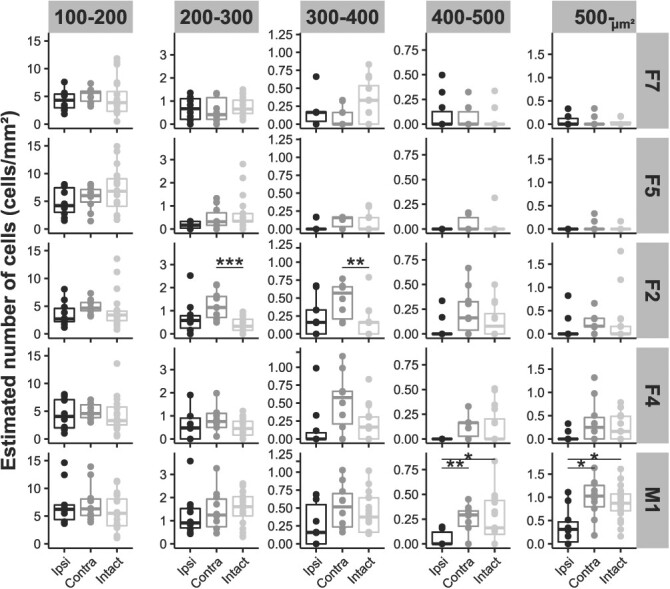
The number of pyramidal neurons in layer V per mm^2^. In the M1, the number of cells/mm^2^ with cell surface area (400–500 and 500– μm^2^) in the ipsi-hemisphere is lower than that in the contra-hemisphere and in intact macaques. Wilcoxon signed-rank test or Wilcoxon rank sum test corrected by the Bonferroni method was performed for the paired group (ipsi/contra) and nonpaired group (intact/ipsi and intact/contra) (^*^P < 0.05, ^*^^*^P < 0.01, and ^*^^*^^*^P < 0.001). The horizontal line in each boxplot represents the median value, and the top and bottom of the box correspond to the 75th and 25th percentile values, respectively. Each dot represents raw data. F7, dorsal–rostral part of the premotor cortex; F5, ventral–rostral part of the premotor cortex; F2, dorsal–caudal part of the premotor cortex; F4, ventral–caudal part of the premotor cortex; and M1, hand area of the primary motor cortex.

Quantitative density analyses of SMI-32-positive neurons indicated that the number of cells/mm^2^ with cell surface area (400–500 μm^2^ and 500– μm^2^) in the ipsi-M1 was lower than that in the contralateral hemisphere as well as in the those of intact macaques (*P* < 0.05, *r* > 0.48, Fig. 6). Our previous analysis using Nissl-stained sections revealed a decrease in the percentage of large neurons in the layer V of the ipsi-M1 following unilateral IC infarcts in macaques (Murata and Higo 2016). The present results reproduced those using the SMI-32 antibody, which specifically stained the subcortically projecting pyramidal neurons in layer V. The density of several cell surface areas (200–400 μm^2^) in the contra-hemisphere of F2 was higher than that in the hemispheres of intact macaques (*P* < 0.01, *r* > 0.54). However, the cell density analysis did not demonstrate significant differences in the F5, where a significant GMV increase was indicated by the whole-brain analysis. Previous studies have revealed dendritic structural changes following stroke (Biernaskie and Corbett 2001; Biernaskie et al. 2004; Papadopoulos et al. 2006; Lee et al. 2020), and that dendritic structures are robustly correlated with GMV changes (Kassem et al. 2013). Thus, we focused on dendritic arborization to identify histological alterations behind GMV changes.

**Fig. 7:**
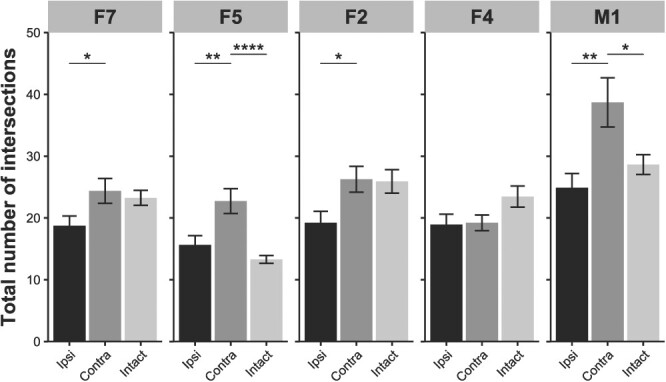
The total number of dendritic intersections with the Sholl circles of layer V neurons. Significant increase in the total number of intersections was observed in the contra-F5 and contra-M1 relative to those in the ipsi-hemisphere and the hemisphere of intact macaques. For the statistical analysis, paired or nonpaired *t*-test corrected by the Bonferroni method was performed for the paired group (ipsi/contra) and nonpaired group (intact/ipsi and intact/contra) (^*^P < 0.05, ^*^^*^P < 0.01, and ^*^^*^^*^P < 0.001). The data represent the mean ± SEM. F7, dorsal–rostral part of the premotor cortex; F5, ventral–rostral part of the premotor cortex; F2, dorsal–caudal part of the premotor cortex; F4, ventral–caudal part of the premotor cortex; and M1, hand area of primary motor cortex.

The total number of intersections with the Sholl circles of layer V neurons in the contra-F7, F5, F2, and M1 hemispheres was significantly higher than that in the ipsi-hemisphere (*P* < 0.05, *d* > 0.33, Fig. 7). Moreover, the number in the contra-F5 and contra-M1 was higher in both areas than that in the hemispheres of intact macaques (*P* < 0.0001, *d* = 0.78 in contra-F5; *P* = 0.0113, *d* = 0.40 in contra-M1), consistent with the results of the ROI-based analysis.

**Fig. 8:**
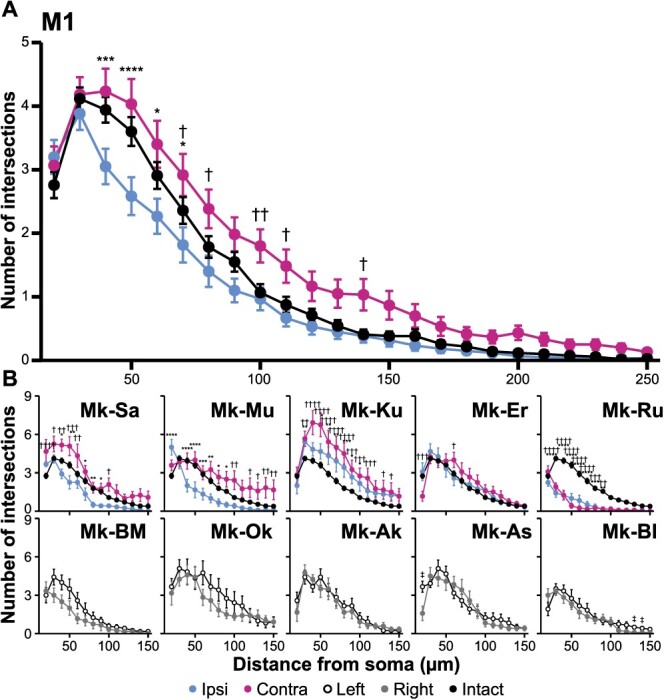
Dendritic arborization of SMI-32-positive neurons in the layer V of the hand area of M1. A) Merged data of Sholl profiles denote that the number of intersections in the ipsi-hemisphere is significantly lower than that in intact macaques at 40–70 μm, whereas the number of intersections in the contra-hemisphere is significantly higher than that in intact macaques at 70, 80, 100, 110, and 140 μm. B) Individual data denote a significant interhemispheric difference in a number of distances in the 3 of the 5 lesioned macaques, compared with little difference in the number of intersections between the hemispheres in intact macaques. For the statistical analysis, 2-way factorial ANOVA with multiple comparisons corrected by Bonferroni has been performed as follows: the ipsi-hemisphere and intact (^*^P < 0.05, ^*^^*^P < 0.01, ^*^^*^^*^P < 0.001, and ^*^^*^^*^^*^P < 0.0001) and the contra-hemisphere and intact (^†^*P* < 0.05, ^†^^†^*P* < 0.01, ^†^^†^^†^*P* < 0.001, and ^†^^†^^†^^†^*P* < 0.0001). For intact macaques, paired *t*-test was conducted between hemispheres depending on the distance from the soma (^‡^*P* < 0.05). The data represent the mean ± SEM.

**Fig. 9:**
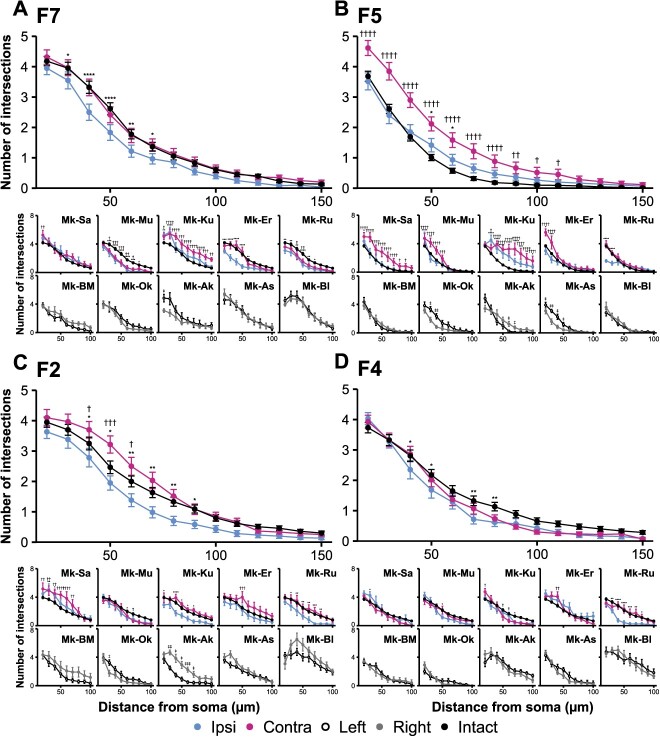
Dendritic arborization of SMI-32-positive neurons in the layer V in the premotor cortices. Sholl profiles are displayed for F7 (A), F5 (B), F2 (C), and F4 (D). Merged data reveal that the number of intersections in the F7, F2, and F4 of the ipsi-hemisphere is significantly lower than those in intact macaques. Merged data in B reveal that the number of intersections in the contra-F5 is significantly higher than that in intact macaques at a distance <110 μm. For the statistical analysis, 2-way factorial ANOVA with multiple comparisons corrected by Bonferroni has been performed as follows: the ipsi-hemisphere and intact (^*^P < 0.05, ^**^P < 0.01, ^***^P < 0.001, and ^****^P < 0.0001) and contra-hemisphere and intact (^†^*P* < 0.05, ^††^*P* < 0.01, ^†††^*P* < 0.001, and ^††††^*P* < 0.0001). For intact macaques, paired *t*-test was conducted between hemispheres depending on the distance from the soma (^‡^*P* < 0.05, ^‡‡^*P* < 0.01, and ^‡‡‡^*P* < 0.001). These data represent the mean ± SEM.

The number of intersections at each distance from the soma in M1 (Fig. 8) in the ipsi-hemisphere was lower than those in the contra-hemisphere in 3 of the 5 lesioned macaques (Mk-Sa, Mk-Mu, and Mk-Ku). However, we observed little difference in the number of intersections between the hemispheres in intact macaques. Relatively larger lesions were made within the IC of Mk-Ru, which displayed a significantly lower number of intersections in the bilateral hemispheres, relative to the mean of the hemispheres in intact macaques. Thus, dendritic degeneration may have occurred in both hemispheres. The merged data demonstrated significantly lower number of intersections in the ipsi-M1, with a maximum difference of 50 μm from the soma (*P* < 0.0001, *d* = 0.43), compared with the mean of hemispheres in intact macaques. Moreover, we observed a significantly higher number of intersections in the contra-M1, with a maximum difference of 100 μm (*P* = 0.0034, *d* = 0.42) than those in intact macaques. The aforementioned results suggested a decrease in dendritic arborization in the ipsi-M1, besides an increase in the contra-M1. Significantly lower numbers of intersections in the ipsi-hemisphere relative to the intact hemisphere were observed in the F7 (maximum difference at 40 μm, *P* < 0.0001, and *d* = 0.39), F2 (maximum difference at 70 μm, *P* = 0.0013, and *d* = 0.40), and F4 (maximum difference at 70 μm, *P* = 0.0013, and *d* = 0.41, Fig. 9), thus suggesting decreased dendritic branching in these premotor cortices. In contrast, we observed a significantly higher number of intersections in the contra-F5, with a maximum difference at 30 μm (*P* < 0.0001, *d* = 0.63), as well as in the contra-F2, with a maximum difference at 50 μm (*P* = 0.0002, *d* = 0.33), thereby suggesting increased dendritic arborization in these areas.

## Discussion

In the present study, macaques subjected to unilateral IC infarcts demonstrated significant increases in both GMV and the dendritic arborization of SMI-32-positive layer V neurons in both the contra-F5 and the contra-M1, suggesting the formation of synaptic connections in these areas. GMV decreases were also indicated by the ROI-based analysis in the ipsi-F2 and F4, besides the consistent decreases in dendritic arborization. The histological analysis revealed the shrinkage of the neuronal cell bodies and dendritic degeneration in the ipsi-M1 and F7 regions, respectively, despite no significant GMV reduction.

### Plastic changes underlying motor recovery

Previous studies have reported a significant GMV increase following stroke; nonetheless, it is not confined to the motor cortex rather involves changes in multiple brain regions outside the motor cortices (Fan et al. 2013; Abela et al. 2015; Cai et al. 2016; Yu et al. 2017; Wang et al. 2018). The principal factor responsible for the inconsistency between previous studies and the present study may be the occurrence of stroke focal to the IC, a brain region specifically dedicated to motor function. Moreover, the variance of lesion extension in this macaque model was smaller than that in stroke patients (Jiang et al. 2017; Wang et al. 2019); the computed coefficient of variation, a parameter on variance of data, was 0.25 for total lesion volume in the present study but over 1 in patient studies (Jiang et al. 2017; Wang et al. 2019). The functional reorganization of the F5 underlies the recovery of motor function in brain-damaged monkeys (Liu and Rouiller 1999; Frost et al. 2003; Hoogewoud et al. 2013; Murata and Higo 2016; Kato et al. 2020) and patients following stroke (Loubinoux et al. 2007; Horn et al. 2016), thus suggesting that changes in the F5 may form a structural basis for functional reorganization in this area.

The present whole-brain analysis revealed maximum GMV increase in the convexity of the contra-F5, the coordinates *x* = 24.0, *y* = 5.0, and *z* = 4.0, that corresponds to the subregion F5c (Belmalih et al. 2009; Kurata 2018). F5c includes mirror neurons that are activated when grasping an object and on observing the same action being performed by another monkey or human (Fabbri-Destro and Rizzolatti 2008) although anatomical connections with the spinal cord are lacking (He et al. 1993; Galea and Darian-Smith 1994). The original function of mirror neurons is presumably to provide the visual feedback needed to shape the own hand for grasping (Oztop and Arbib 2002), and this system might be recruited during recovery of hand motor functions (Bonstrup et al. 2018). Our previous study reported that positron emission tomography showed enhanced activation in the F5c after the M1 lesion during periods of motor recovery (Murata et al. 2015). Furthermore, another study in the macaque model of IC infarcts revealed that temporal inactivation of the region corresponding to F5c induced motor deficits after recovery (Kato et al. 2020), suggesting the contribution to functional recovery. The GMV increase in F5c revealed in the present study may provide a structural basis for the functional changes that occur in this region during motor recovery after brain damage although histological analysis in each F5 subregion is necessary to reinforce this assumption.

Our findings suggested structural changes in the F5 in the contra-hemisphere but not in the ipsi-hemisphere. Functional reorganization of the motor cortex occurs in both hemispheres ipsilateral and contralateral to stroke; the ipsilesional hemisphere is dominantly activated in motor recovery for mild stroke lesion, whereas the contralesional hemisphere is activated for severe stroke lesion (Johansen-Berg et al. 2002; Schaechter and Perdue 2008; Bestmann et al. 2010; Rehme et al. 2011; Bradnam et al. 2012; Kantak et al. 2012; Bajaj et al. 2016; Touvykine et al. 2016; Dodd et al. 2017; Kato et al. 2020). Despite the stroke being focal, the present macaque model of IC infarcts may correspond to patients with severe stroke lesions. This is because the stroke lesion in the IC severely impaired motor output from the affected hemisphere. Therefore, the contra-F5 may have played an essential role in motor recovery in the macaque model. However, structural changes supposedly not associated with GMV changes may occur in the ipsi-F5. Studies using the monkey model of M1 lesions reported the development of efferent neural projections from the aforementioned area during motor recovery (Dancause et al. 2005; Yamamoto et al. 2019). Moreover, our findings suggested structural changes in the contra-M1, with reports of the dendritic growth of layer V pyramidal neurons during motor recovery following stroke in previous rodent studies (Biernaskie and Corbett 2001; Biernaskie et al. 2004; Papadopoulos et al. 2006). Therefore, the functions and structures of the contra-M1 may change in conjunction with those of the F5 during motor recovery. This warrants further studies on the changes in connections originating from the motor cortices, using projection measurement technologies such as diffusion tensor imaging (DTI) and anatomical tracer analysis to clarify structural changes in neuronal network underlying motor recovery following brain damage.

### Degenerative alterations

The decrease in the percentage of large neurons in the ipsi-M1 was consistent with previous findings in rat and macaque models of IC infarcts, which demonstrated degenerative changes in neurons in the motor cortex (Murata and Higo 2016; Lee et al. 2020). Endothelin-1 was injected into the posterior IC, where neuronal pathways originating from the hand area of M1 pass through; thus, the decrease may be attributed to retrograde degeneration following axonal damage (Duering et al. 2015; Wei et al. 2019; Cheng et al. 2020). In contrast, contra-F2 showed an increase in the number of pyramidal neurons with medium cell body size. Although the reason is unclear, it may reflect structural changes underlying functional compensation because this area was reported to be more active during the movement of the affected hand (Ward et al. 2003) and exert a functionally relevant causal influence on motor output in stroke patients (Bestmann et al. 2010).

Individual difference was observed in cortical changes, and it is probably due to the difference in the extent of the subcortical lesion among subjects because the location and volume of the lesion are known to affect severity of retrograde degeneration (Wannier et al. 2005; Higo et al. 2009; Murata and Higo 2016). Unlike the trend in the other macaques, Mk-Er and Mk-Ku had increased or no change in the number of dendritic intersections in the ipsi-M1 compared to that in the intact macaques. This may be because these macaques had relatively small lesions and might have had dendritic degeneration with subsequent dendritic regrowth in the ipsi-M1. As previously reported, dendritic structures in the peri-infarct area decreased once and reverted to the level equal to or more than that in control after infarcts (Brown et al. 2007; Brown et al. 2008; Murphy and Corbett 2009; Wu et al. 2014; Ji et al. 2021), and compensatory dendritic changes are known to occur in the cortical regions more proximal to the lesion in less damaged individuals (Contestabile et al. 2018). In contrast, Mk-Ru with a larger lesion within the IC displayed bilateral dendritic decrease compared to that in intact macaques. This is probably because of secondary or tertiary transsynaptic degenerations. For further analysis, DTI can be beneficial for elucidating the changes in structural networks between hemispheres.

Moreover, we observed dendritic degeneration in the F7, F2, and F4 in the ipsi-hemisphere. Secondary or tertiary transsynaptic degeneration following the degeneration of M1 neurons may also be involved in dendritic degeneration in the premotor cortices. Furthermore, axons originating from these premotor cortices were directly affected by endothelin-1 injection. This is because our previous study demonstrated that the cerebral edema transiently expands outside the posterior IC, where projections from the M1 exist several days post infarction (Murata and Higo 2016). The damage of axons originating from these premotor cortices may also affect the plastic changes observed in the contra-F5. To elucidate which motor fibers were injured by infarcts, we further need to investigate the changes in the descending pathways originating from motor cortices using DTI.

### The relationship between dendritic alteration and GMV changes

Our findings in the contra-F5, M1, and ipsi-F2 and F4, with dendritic arborization changes consistent with the ROI-based GMV changes, are in line with a previous study combining MRI and histological analysis in rodents, which reported a positive correlation between the GMV changes and dendritic structural changes (Kassem et al. 2013). The GMVs of the ipsi-M1/F5 and contra-F5 showed significant positive correlation with the number of dendritic intersections in layer V (*R*^2^ > 0.8, *R* > 0.9, *P* < 0.05) although significant correlation was not found in the other areas examined (data not shown). In contrast, a significant positive correlation between GMV and total count of pyramidal neurons in layer V was only found in the ipsi-F5 (*R*^2^ = 0.89, *R* = 0.96 and *P* = 0.00968). Therefore, the number of dendritic intersections showed a higher correlation with GMV than the number of pyramidal neurons, and this may be attributed to the fact that dendrites mainly account for GMV components (30%) in contrast to neuronal soma (7.8%) (Bennett 2011). Moreover, anatomical features other than layer V probably affect GMV, and this may be a reason that significant correlation was not found between GMV and the number of dendritic intersections in some areas. In addition, the present sample size was possibly insufficient for reflecting the relationship among parameters.

The present whole-brain analysis could not detect GMV changes in the ipsi-F2, F4, and contra-M1 where significant changes were observed in the ROI-based analysis. The inconsistency between the whole-brain and ROI-based analyses may be because the former yielded more conservative results due to a strict threshold and multiple corrections. However, as statistical tests are conducted per voxels, a cluster being composed of voxels with functional similarity can be detected in the whole-brain analysis. Although the present ROI-based analysis is more liberal in that no multiple correction was employed, it requires relatively consistent changes across the ROI. Additionally, the whole-brain analysis did not detect significant GMV reduction in the ipsi-M1 and F7, despite degenerative changes in the neurons, including the shrinkage of neuronal cell bodies and dendritic degeneration. Nevertheless, a correlation analysis between IC lesion volume and GMV changes indicated a significant negative correlation in the ipsi-M1, thus suggesting that degenerative changes depending on the IC lesion volume were reflected in the present whole-brain analysis. Taken together, the detection power of the present whole-brain analysis was lower than that of the histological analysis. This may be attributed to our small sample size (*n* = 5); macaque VBM studies typically include smaller sample than human VBM studies for ethical reasons (Quallo et al. 2009; Nagasaka et al. 2021). The low detection power of the present whole-brain analysis may also be attributed to the fact that the proliferation of glial cells may counterbalance the degenerative changes of neurons. This is because prior studies have demonstrated increases in both astrocytes (Nam et al. 2020) and microglia (Kato et al. 2021) in the motor cortex following IC infarcts, and because the transient swelling of astrocytes is associated with GM expansion (Schmidt et al. 2021). Furthermore, changes in synaptic number and structures have been shown to be involved in experience-dependent plasticity (Clark et al. 2019; Schmidt et al. 2021) and functional recovery after infarcts (Streffing-Hellhake et al. 2020); therefore, they may also affect GMV in the present macaque model. Future studies investigating changes in glial cells and synaptic densities in parallel with GMV changes are needed to understand the full picture of the changes that occur in the nervous system after brain damage.

## Data availability

Data reported in this manuscript are available from the corresponding author on reasonable request.

## Funding

This work was supported by KAKENHI (No. 20H04061) from Japan Society for the Promotion of Science and a Grant-in-Aid for Scientific Research on Innovative Areas “Hyper-adaptability for overcoming body-brain dysfunction: Integrated empirical and system theoretical approaches” (No. 20H05490) from Ministry of Education, Culture, Sports, Science and Technology, Japan.

*Conflict of interest statement*: None declared.
